# Eumycetoma Osteomyelitis Calcaneus in Adolescent; report of case and literature review

**DOI:** 10.1186/s12879-021-06695-3

**Published:** 2021-09-23

**Authors:** Ammar Awad, Adnan Alnaser, Hozifa Abd-elmaged, Reyad Abdallah, Hussam S. Khougali

**Affiliations:** 1 University of Kordofan, Al-Ubayyid, Sudan; 2 Omdurman Teaching Hospital, Khartoum, Sudan; 3 Khartoum North Teaching Hospital, Khartoum, Sudan; 4 University of Gezira, Wad Medani, Sudan

**Keywords:** Mycetoma, Osteomyelitis, Surgical debridement, Fungal infection

## Abstract

**Background:**

Mycetoma is the most common neglected disease in humans. It is a chronic, progressive, and destructive disease primarily caused by fungi or bacteria characterized by formation of dark pale grains commonly involve skin, soft tissue and rarely bone.

**Case presentation:**

A 19 year old male patient with chronic right ankle pain, swelling and abscess formation for more than 1 year, patient was treated repeatedly with incision and drainage without any success. No X-ray, biopsy or swab for culture and sensitivity had been considered through the course of presentation. Patient was referred to Omdurman hospital where osteomyelitis secondary euomycetoma infection has been confirmed based on radiological and pathological assessment. Patient was treated surgically with aggressive debridement and bone curettage plus postoperative Itraconazole for 1 year.

**Conclusion:**

Clinicians must consider osteomyelitis as important differential diagnosis during initial assessment Eumycetoma infection in adults. Aggressive bone curettage followed by regular X-ray follow up can be limb saving procedure in such cases.

## Background

Mycetoma defined as a chronic cutaneous and subcutaneous swelling caused by two types of organisms, either fungus type (eumycotic) or bacterial type (actinomycotic) [1]. It considers the most common neglected disease of human in African [1, 2]. It involves the skin, soft tissues and rarely bones. Formation of dark pale grains, poor response to treatment and high recurrence rate are the most common characteristic features of this disease [2, 4]. The disease is endemic in Africa forming area like belt known as mycetoma belt countries, the highest incidence has been recorded in Sudan [3, 4]. The chronic inflammatory granuloma, numerous deformations, disabilities and high morbidity rate are the commonest known complications of mycetoma, the disease can be potentially fatal in its late stage [5, 6]. Clinically, mycetoma starts as a small painless subcutaneous nodule which gradually increases in size, then multiple sinuses with seropurulent discharge and eventually multicolored grains appear [7]. Clinical, radiological and histopathological assessment of such lesion is deemed important for accurate diagnosis and management of especially when osteo-articular infection is suspected. Treatment of mycetoma depends mainly on the etiological agent, site of infection, and extent of the disease [8]. This review is to highlight and demonstrate the destructive features of bone involvement by mycetoma in adult patients that usually associate with incomplete clinical and pathological assessment of this lesion and the impact of delayed presentation or late diagnosis to the overall outcome.

## Case presentation

**Fig. 1:**
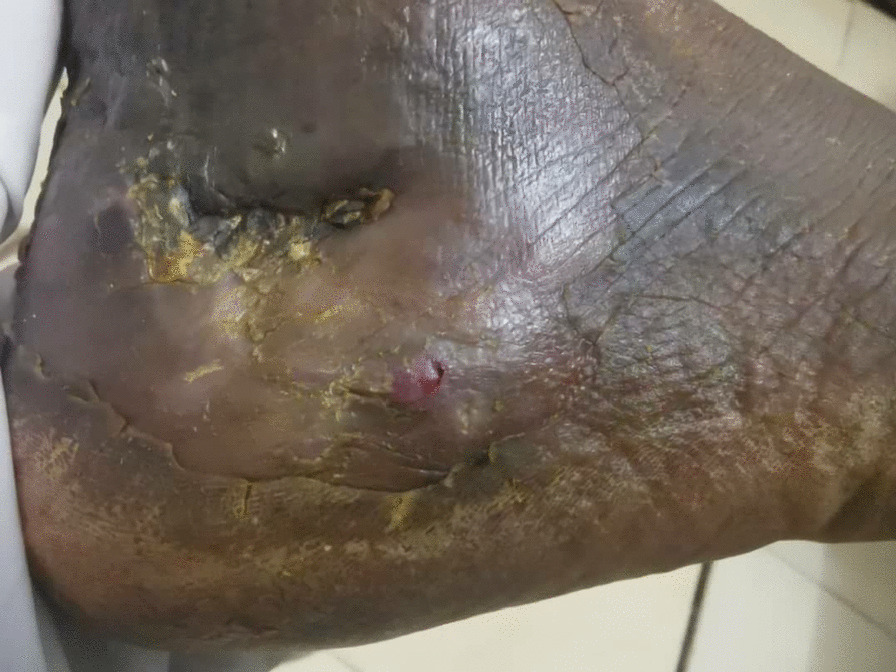
Sinus formation with purulent pale discharge and black grains

**Fig. 2:**
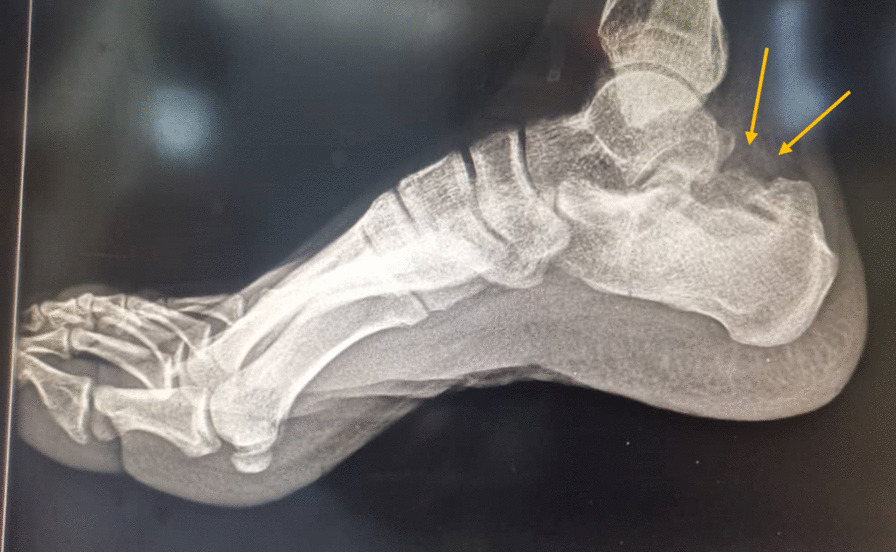
lateral view X-ray Right foot, shows features of cortical erosion and central cavitation consistent with chronic osteomyelitis in the calcaneum bone

**Fig. 3:**
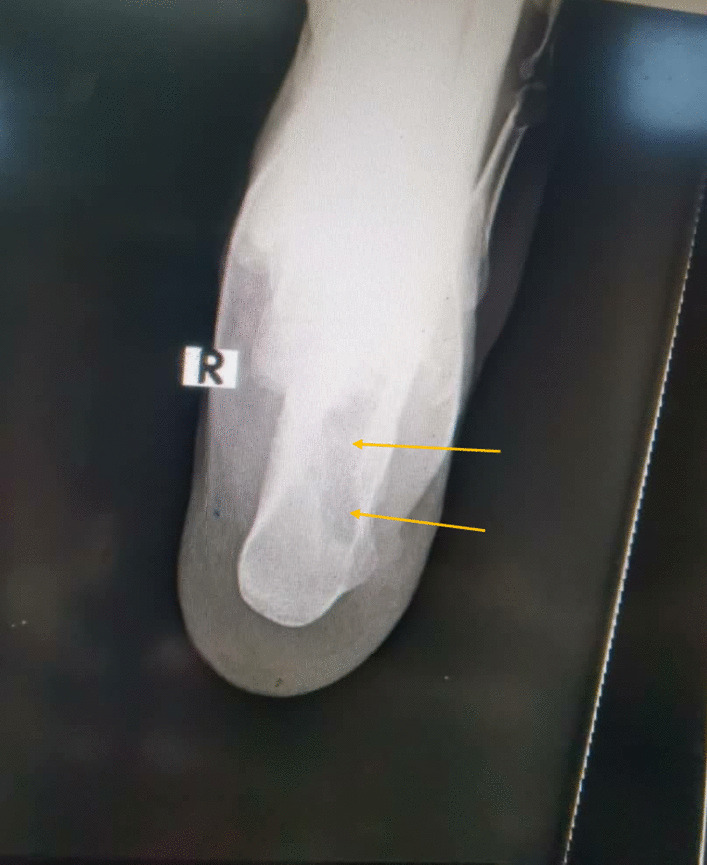
AP view X-ray revealed calcaneus scalloping lesion forming 2 cavities posterior to the posterior facet of the calcaneus consistent with chronic osteomyelitis

**Fig. 4:**
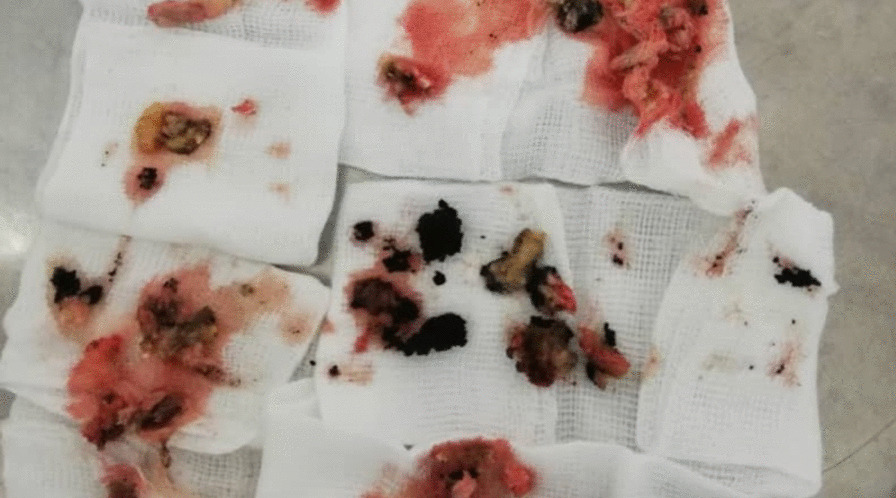
image shows intraoperative finding and some black grains excised from the bone during curettage

A 19 year old male farmer from rural area in Sudan presented to our clinic at Omdurman Hospital –Sudan, complaining of chronic ankle pain, swelling and abscess formation for one year, it started with small painless swelling that gradually increasing in size, over period of time his life style had been affected by increasing pain intensity, walking difficulties and inactivity, symptoms became more severe when limited restriction of movement involved the subtalar joint. Sinus formation with purulent pale discharge and black grains were noted. Initially and without proper assessment, he was diagnosed with cellulitis and abscess formation, thus he was treated accordingly by incision and drainage. No biopsies or swabs had been taken for culture and sensitivity. Post-operative course oral antibiotic has been prescribed for 7 days. Since then the deterioration in his general condition had been growing steadily. Painful limping, swelling and limitation of movement on the affected side had been worsening dramatically. Furthermore he ended by using crutches. On clinical examination there was obvious ankle swelling and tenderness mainly at the lateral side of the hind foot, single sinus with active greenish discharge was identified [Fig. 1]. Hematological investigations were unremarkable, X-ray revealed calcaneus scalloping lesion forming 2 cavities posterior to the posterior facet of the calcaneus consistent with chronic osteomyelitis [Figs. 2, 3]. Surgical debridement under spinal anesthesia and tourniquet was employed in lateral decubitus position with lateral extensile calcaneus approach. Debridement was done and the 2 cavities were cleaned with aggressive curettage, the black grains consist of eumycetoma were confirmed by histopathology [Fig. 4]. Hence, Itraconazole 400 mg daily for at least 1 year was prescribed. Six months follow up as it shown in the Table 1 below:

**Table 1:** Six months follow up

Time	Event
1 month visit	Wound healing was good, no signs of early postoperative complication X-ray no evidence of recurrence
2 month visit	Patient very satisfied with final postoperative results X-ray no evidence of recurrence
3 month visit	Bloods was unremarkable X-ray no evidence of recurrence
4 month visit	Blood test was unremarkable X-ray no evidence of recurrence
5 month visit	Wound healed completely Blood test unremarkable X-ray no evidence of recurrence
6 month visit	X-ray no evidence of recurrence Walking without crutches

## Discussion and conclusion

Eumycetoma Osteomyelitis Calcaneus is very rare condition to be reported in adult patient. Destructive bone features by mycetoma species and formation of grains in situ are extremely rare findings to be seen within the bone, when it happens the risk of lower limb loss by amputation as an option of treatment will be very high. Painless nature of the lesion, low socioeconomic status and lack of awareness are the common reasons drive Sudanese patients to present late.

As mentioned above the treatment of mycetoma depends mainly on the etiological agent, site of infection and extent of the disease [8]. Until recently in Sudan, the only available treatment for mycetoma was amputation, as no therapeutic consensus has been reached. Actinomycetoma (bacterial type) is usually treated with medications only as it shows relative response to medical treatment. For eumycetoma (fungal type), a combination of medical treatment (anti-fungal agents) and various surgical excisions are the gold standard as this type usually very resistant to solely medical treatment [9].

According to the New Radiographic Classification of Bone Involvement in mycetoma by Mohamed E. Abd El Bagi, our patient radiographs show soft tissue involvement, cortical erosion, and central cavitation of solitary bone (calcaneus) which would be classified as class 3 [9]. This classification can be useful to determine which is the best surgical option can be offered to the patient. Cortical erosion and central cavitation are commonly seen in patients with Eumycetoma Osteomyelitis, revision of X-ray by orthopedic surgeon or radiologist is always recommended in such cases to minimize the rate of the misdiagnosis.

Eumycetoma causative agents are difficult to ascertain. Hence, assessment should include full pathological analysis of the affected area with fine needle aspiration cytology and histopathology to build solid diagnosis. A Tru-Cut needle biopsy and immunohistochemistry are now strongly recommended to avoid problems of inadequate specimens commonly associated with incisional biopsy [10]. With clear identification of underlying causative organism both prognosis and management outcome can be improved significantly.

Unfortunately since eumycetoma has had a poor response to medical therapy, surgical approaches are all that available. Many Sudanese patients undergo many operations with several regimens of ketoconazole and itraconazole to enable better response. However, surgical options for mycetoma treatment in Sudan range from wide local excision to amputation of the affected part, the balance between wide local excision and bloodless field during operation are deemed essential to achieve good surgical outcome [11].

The postoperative recurrence rate varies from 25 to 50%, the Predictors of post-operative mycetoma Recurrence depends on age, duration, site of involvement and no previous history of mycetoma. Thereby, surgical operation considers the lowest risk of recurrence [12].

Surgical intervention usually associates with high rate of morbidity and disability among mycetoma patients in Sudan. Post-operative wound care, physiotherapy and adherence to antifungal agent are mandatory for better surgical outcomes and to avoid the joint stiffness, bone deformities and eventually disabilities. Peri-operative and post-operative antibiotics with good dressing techniques are needed to minimize the high rate of associated complications.

In conclusion, Eumycetoma Osteomyelitis Calcaneus in Adolescent is extremely rare condition. Clinicians must consider osteomyelitis as a differential diagnosis when they are dealing with eumycetoma infection. Triple assessment (Clinical examination, CT scans and Histopathology) is deemed important to assess bone involvement in patients with euomycetoma, aggressive bone curettage followed by regular X-ray follow up can be limb saving procedure in such cases. Generally the treatment of mycetoma osteomyelitis is case—by—case according to the predictors of post-operative recurrence.

## Data Availability

The data used in this report is available to readers.
